# A new algorithm Precision OncoPanels (PrOPs) identifies short individualized actionable panels that can guide cancer treatment: a pan-cancer analysis of TCGA cohorts

**DOI:** 10.1093/nargab/lqaf177

**Published:** 2025-12-08

**Authors:** Shrisruti Sriraman, Debajyoti Das, Nagasuma Chandra

**Affiliations:** IISc Mathematics Initiative, Indian Institute of Science, Bangalore 560012, India; Department of Biochemistry, Indian Institute of Science, Bangalore 560012, India; IISc Mathematics Initiative, Indian Institute of Science, Bangalore 560012, India; Department of Biochemistry, Indian Institute of Science, Bangalore 560012, India; Department of Bioengineering, Indian Institute of Science, Bangalore 560012, India

## Abstract

Precision oncology, enabled by next-generation sequencing (NGS), has shown tremendous potential for use in a clinical setting for cancer diagnosis and treatment. The biggest promise is to make treatment more precise and tailored for individual patients, departing from the one-size-fits-all approach. However, the translation of genomic panels into clinical practice and their wider implementation are met with challenges. Currently, only those patients who have frequently observed mutations in that cancer benefit from the NGS approach. There is an urgent need to expand the scope of this to all patients, for which new methods are required to be developed so as to identify key actionable gene panels in all patients. We address this need and present a new algorithm, PrOPs (Precision Onco Panels), that identifies short actionable driver panels by integrating genomics, transcriptomics, genome-wide protein–protein interactions, and precision network construction and analysis. We tested the algorithm on 2180 patients from six cancer types from TCGA (BRCA, COAD, GBM, LIHC, LUAD, and SKCM) and predicted patient-specific cancer driver genes. PrOPs outperforms the existing network-based methods that identify personalized drivers and also capture rare and patient-specific cancer drivers. Among the clinical cohorts, PrOPs identified clinically relevant actionable panels in 93% of patient cases. The extensive testing of our algorithm and demonstrated generalizability in six different cancers indicate the usefulness of our algorithm in precision oncology.

## Introduction

One of the major causes of cancer is genomic alterations such as gene mutations and copy number variations [1]. Next-generation genomics sequencing (NGS) technologies are increasingly providing a wealth of data on patients’ whole-genome, whole-exome, and transcriptome in a variety of cancers and have revolutionized the field of cancer genomics [2]. In particular, it has led to significant advances in the understanding of disease mechanisms, as well as in shaping new ways of disease diagnosis, patient subtyping, estimating disease prognosis, and selecting optimal treatment strategies, overall pushing the frontiers of precision medicine [3]. The power of NGS in cancer diagnosis and treatment is best leveraged when implemented in a consolidated digital health framework containing modules for genomic profiling of tumours, analysis and interpretation of the genomic data to provide clinical decision support, evidence-based treatment selection, and patient monitoring to determine the efficacy of treatment. While there have been tremendous advances on the technological front, making genomic profiling feasible and panels identified, the wider translation of genomic panels into clinical practice is actually challenged by the complexity of the panels themselves. A pivotal element in this sequence of steps is the interpretation of the genomic data for each patient.

Currently, only those patients who have variations in the signature genes in their panels for which targeted therapies are available benefit from this approach. The problem is that only a small percentage of patients carry such signature variants, and it is increasingly becoming clear that there are a large number of other variants constituting disease driver events. With rapid advances in the number of approved targeted therapies available for use, the number of actionable genes is on the rise. The bottleneck then is in the identification of key variants in each patient. The concept of driver gene mutations in cancer has long been established, with numerous methods developed to distinguish drivers from passenger genes [4]. It is amply clear that the disease is typically polygenic in nature, and each patient presents a unique genetic landscape, influenced by multiple factors such as genetic predisposition, environmental exposures, epigenetic modifications, disease stage, rate of progression, and the patient’s overall health status. The driver mutations do not occur in isolation in the patient but often along with hundreds of other mutations, which together lead to the emergent behaviour that is the disease. Often, the combined effect of variations in different genes is much larger than the effect of changes in individual genes. However, the subtyping that is typically carried out is based on frequently occurring driver mutations based on a cohort or population level that is studied. This heterogeneity manifests in diverse molecular profiles as well as disease and treatment outcomes among individuals with apparently the same cancer type. This makes it inadequate to rely solely on signature driver mutations. The problem is further highlighted by the fact that therapies targeting individual mutations are observed to be refractory to treatment in several cases, owing to factors such as co-existing mutations in other genes that result in alternate tumorigenesis mechanisms [5, 6]. Thus, the identification of the full component of the set of key driver mutations, rather than individual driver genes by themselves, is required for identifying clinically actionable decisions in terms of selecting personalized therapies with high efficiency.

A multitude of methods have been developed in recent years for identifying driver mutations in cohorts and populations [7–14]. The basic approach used in these methods involves a comparison of the individual mutation rates with that of the background rate and the detection of genes that are recurrently mutated. These methods are broadly based on analysing data from large patient datasets and detecting frequently occurring mutations using statistical methods and, recently, also machine-learning-based methods. Methods that consider transcriptional variations and knowledge-driven pathways have been developed recently and are seen to be more promising in elucidating driver mutations. Machine learning has better accuracy but still only identifies individual mutations frequently occurring in cohorts or populations and misses out on combinations of drivers which are highly variable in individual patients. A few methods have been developed in recent years for identifying patient-level panels, DriverNet [15], Prodigy [16], DawnRank [17], NetBox [18], TieDIE [19] being some examples. All of them typically identify panels containing multiple genes, making it difficult to select the most important among them for actionability. This can be overcome if short panels of personalized influential drivers can be identified in each patient.

We address this in this work and develop PrOPs, a method to identify short driver gene panels in each individual. We demonstrate the usefulness of our method in ~2180 patient samples belonging to 6 different cancers. Our method is based on combining whole exome data, transcriptome data, and a knowledge-based protein–protein interaction network, comprehensively capturing genome-wide structural as well as functional interactions. We benchmark our method against two recent methods that have a similar goal and show superior performance of our method overall. We find that nearly $93\%$ of patients have at least one actionable gene among their panel genes.

## Materials and methods

### Dataset description

Six cancer cohorts - Colon Adenocarcinoma (COAD), Glioblastoma (GBM), Breast Invasive Carcinoma (BRCA), Lung Adenocarcinoma (LUAD), Liver Hepatocellular Carcinoma (LIHC), and Skin Cutaneous Melanoma (SKCM) - were downloaded from the publicly available TCGA. All these datasets are from the TCGA Firehose legacy collection. These were selected to cover a wide range of reported mutational burden, the availability of both whole-exome and transcriptome data, as well as a range of sample sizes, and the availability of clinical phenodata such as staging and survival. Mutation profiles were downloaded from the GDC repository portal [20] for all these cohorts (full description of the data is in Table 1). Hypermutated patient samples with >1000 mutations were excluded from the analysis based on [4].

**Table 1.** Dataset description of the tumour and non-tumour control samples of the six cancer cohorts used in the study

Cohort	Number of samples		Exome	Transcriptome
	Tumour	Non-tumour (control)		
Breast Adenocarcinoma (BRCA)	965	113	Yes	Yes
Colon Adenocarcinoma (COAD)	142	41	Yes	Yes
Glioblastoma (GBM)	147	5	Yes	Yes
Liver Hepatocellular carcinoma (LIHC)	365	50	Yes	Yes
Lung Adenocarcinoma (LUAD)	229	59	Yes	Yes
Skin Cutaneous Melanoma (SKCM)	332	1	Yes	Yes

This study included patients who had both exome and transcriptome data available from the TCGA Provisional data.

### Gene expression analysis

Raw htseq-counts were downloaded from the GDC repository portal [20] for each sample and used as input for EdgeR [21] to identify differentially expressed genes. The raw counts were corrected for the gene length and normalized using geTMM [22]. The counts were fitted to the Generalized Linear Model (GLM), and glmFTest, implemented in the EdgeR package, was used to identify the differentially expressed genes for each patient sample. Standard parameters, depending on library size and dispersion range, were used for all cohorts except SKCM. In the case of SKCM, since only one healthy control was available, an exact test was used for fold change calculation with an assumed dispersion of 0.4 (according to the EdgeR pipeline). Genes with $\log _{2}\text{FC} \ge 1 \ \text{or} \ \log _{2}\text{FC} \le -1$ and an FDR $\le$ 0.05 were considered as DEGs.

### Contextualization of patient networks and identification of *Mutpaths*

A genome-wide knowledge-based protein–protein interaction network was used in this study. We used the master human protein–protein interaction network, hPPiN2, curated earlier in our laboratory consisting of 20183 nodes (genes) and 255486 edges, corresponding to interactions between the proteins [23]. The network was contextualized by integrating with the transcriptome data, and individual networks were generated for each patient.

The transcriptome data obtained from TCGA were integrated with the network by weighting the nodes. Each node was weighted (NW) proportional to the fold change in its gene expression value with respect to its control in Equation (1).


(1) \begin{eqnarray*}
\mathrm{ NW}_{i} = \frac{A_{i}}{B_{i}}
\end{eqnarray*}

where $A_{i}$ refers to the expression of the gene in a specific sample and $B_{i}$ is its expression in the control condition used as reference. Edge weights reflect the extent of interaction between the pair of nodes forming that edge in Equation (2). The edge weight $\mathrm{ EW}_{i, j}$ of two interacting genes ‘*i*’ and ‘*j*’ is calculated as the inverse product of the node weights of the participating nodes:


(2) \begin{eqnarray*}
\mathrm{ EW}_{i,j} = \frac{1}{\sqrt{\mathrm{ NW}_{i} \times \mathrm{ NW}_{j}}}
\end{eqnarray*}

A network mining approach, ResponseNet, previously developed in our laboratory [23, 24], was used to generate individualized networks for each patient, which captures the top-ranked perturbations in the disease samples, as compared to their adjacent normals. Briefly, the ResponseNet algorithm computes path costs and ranks the path based on the activity level through the path. The edge between two nodes is weighted as per Equation (2) to reflect the ‘cost’ of the flow of information. For each patient, the shortest paths with a minimum path length of 3 were computed with Dijkstra’s algorithm with all mutated nodes in that patient as source nodes and all other nodes in the network as sink nodes. A path cost was computed for each shortest path as per Equation (3), where *n* is the path length or the number of edges present in the path, with a minimum value of 3 to a maximum value of ${p}$. The edge weight is computed as the inverse product, such that the highly perturbed edges are selected by the algorithm as the least-cost shortest paths or the highly perturbed biological path.


(3) \begin{eqnarray*}
\mathrm{ Path \, cost} = \sum _{n=3}^{p} \mathrm{ EW}_n
\end{eqnarray*}

A summation of all the edge weights contained in that path is taken as the path cost. The shortest paths were ranked using the path cost, and the top 0.01% of paths were taken to constitute the top response paths (*Mutpaths*). These paths arise from the effects of mutated genes in the disease state on the expression of downstream genes, thereby forming an influence network that serves as the basis for further analysis.

### Max set coverage

A minimal set of mutated genes that covers a maximum number of downstream perturbed genes in the influence networks was identified using the maximum set cover algorithm. The number of DEGs in the *Mutpaths* was considered as the universe. The *Mutpaths* driver genes were considered as sets, and the DEGs that could be reached within four hops were considered as the set cover elements for those driver genes. The driver gene combination was identified for each individual based on the 70% coverage of the DEG universe. This constitutes the *iPanels*. Each driver gene was given a Netscore based on the number of DEGs present within a distance of 4 from its node as represented in Equation (4):


(4) \begin{eqnarray*}
{\mathrm{ Netscore\;of\;a\;driver\;gene} = \sum _{n=1}^{4}\frac{\mathrm{ No.\;of\;DEGs\;in\;the\;\mathit{ n}th\;distance}}{n} }
\end{eqnarray*}

The network analysis was carried out in Python 3.8.10 using the NetworkX package (version 2.6.3) [25].

### Evaluation of candidate driver genes with gold-standard gene sets and benchmarking with other methods

The predicted candidate driver genes were evaluated as a function of overlap with known gold-standard cancer genes. The gold-standard gene list consists of 3347 literature-curated high-confidence genes listed in NCG 7.0 (Network of Cancer Genes) [26], of which 591 are canonical genes and 2756 are candidate drivers across all cancer types. The canonical driver genes in NCG 7.0 are manually curated as a consensus from the three resources—Vogelstein *et al.* (2013) [4], Cancer Gene Consensus by Futreal *et al.* (2004) [27], and Saito *et al.* (2020) [28]. Additionally, tissue-specific driver genes from the Cancer Gene Consensus [27] and CGI [29] were incorporated as gold-standard genes for each of the six cohorts (see Supplementary data). These lists largely overlapped with the NCG genes, with only a few cohort-specific exceptions. Ultimately, this produced six gold-standard gene lists, with an average of 3345 genes. The overlap among these lists highlights genes associated with common cancer hallmark pathways across various tissues and organs.

Driver genes identified by other methods, viz. PRODIGY and DawnRank were compared based on the information retrieval measures: precision in Equation (5), recall in Equation (6), and F1-score in Equation (7).


(5) \begin{eqnarray*}
\mathrm{ Precision }= \frac{\mathrm{ Mutated\;genes\;considered}\cap \;\mathrm{ Gold\;standard\;gene\;list}}{\mathrm{ Mutated\;genes\;considered}} \\
\end{eqnarray*}

(6) \begin{eqnarray*}
\mathrm{ Recall }= \frac{\mathrm{ Mutated\;genes\;considered}\; \cap \;\mathrm{ Gold\;standard\;gene\;list}}{\mathrm{ Gold\;standard\;gene\;list}} \\
\end{eqnarray*}

(7) \begin{eqnarray*}
F1 \mathrm{ score }= \frac{2 \times \mathrm{ Precision }\times \mathrm{ Recall}}{\mathrm{ Precision }+ \mathrm{ Recall}}
\end{eqnarray*}

Using individual *iPanels*, we also obtain a cohort-level list of driver genes for each cancer. This is achieved using a Condorcet ranking that uses the votesys library in R [30].

### Survival analysis

To examine the association between the mutated gene and the combinations of mutations with patient survival, a univariate Cox proportional hazard model was built, and the hazard ratio was estimated. The analysis was carried out using the survival package in R [31].

### PrOPs Risk Score calculation

PrOPs individual Risk Score (PiRS) was formulated to capture the effect of the combination of driver genes on the survival of the patients. The multivariate Cox proportional hazard model was used to calculate the hazard ratio (HR) for each *iPanel*. The prognostic risk score for each individual is calculated as the summation of the product of the Hazard ratio and Net score of each gene in their *iPanel* in Equation (8).


(8) \begin{eqnarray*}
\textstyle \mathrm{ PiRS_i }= \sum _{a=1}^{n}(\mathrm{ Net\;score\;of\;gene}_a \times \mathrm{ HR\;of\;gene}_a)
\end{eqnarray*}

where ${i}$ stands for an individual, ${a}$ refers to the gene in their *iPanel*, ${n}$ is the total number of genes in that *iPanel*, and HR is the Hazard Ratio calculated for that given gene.

### Actionability analysis

A workflow was developed to determine the actionability status of genes in the *iPanels*, using resources from the COSMIC actionability list (version 10) [32]. Information regarding the association of the *iPanel* genes with known drugs, drug combinations, and trial status was retrieved through the DMD (drug–mutation–disease) principle of COSMIC. In addition, our actionability workflow analysed (i) the expression status of each gene in each panel, (ii) their mutation impact, and (iii) associated drugs where present, along with their mode of action (inhibitory or activating) through the gene–drug interactions from DGIdb [33] (last accessed in December 2023).

### Run-time statistics

The PrOPs pipeline is made available on the GitHub repository and requires libraries from Python and R. A sample run using this is described in Supplementary document 2. The time taken to analyse one patient sample on a system with 32 GB RAM and 8 cores was 15 min.

## Results

### PrOPs: overview and algorithmic design

**Figure 1.**
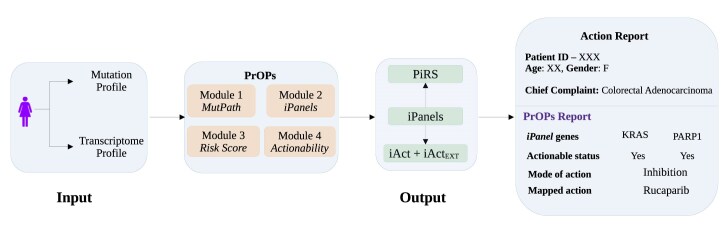
Overview of the PrOPs algorithm. For every individual patient, PrOPs takes their mutation and gene expression profiles as inputs to compute the driver gene panels. PrOPs majorly consists of four modules: (i) Computation of a patient-specific response network called the *Mutpaths* that contains functionally active biological pathways in that patient, (ii) enumeration of *iPanels* that are a set of influential mutated genes identified as driver panels for the individual, (iii) derivation of an individual risk score (PiRS) to understand the risk effect of the panel genes in the disease, and (iv) assessment of the actionability status of the panel genes. These outputs are analysed and consolidated in the form of a report that suggests treatment strategies for each patient.

We present a new algorithm PrOPs, which takes in as input (i) a list of mutations from the exome sequence and (ii) a list of DEGs from the corresponding transcriptome sequence in each patient and outputs (a) a patient-wise short panel of driver mutations (*iPanel*) and (b) tagged with ‘actionability’ as well as suggested ‘actions’ from currently available options (Fig. 1). We showcase the performance of PrOPs in 2180 patient samples from 6 different cancers. We benchmark our algorithm against two widely used network-based algorithms, DriverNet and PRODIGY, available for the same purpose and show that our panels contain ‘key drivers’ and are significantly more compact than those obtained from the others. We compute an individual’s risk score (PiRS) for each patient, which aids in the prediction of the effect of the driver mutations. We further analyse the effect of the mutation of each gene in the *iPanel*, place them in an actionability matrix to derive an actionability category, and map them to available treatment options in all six cancers. Overall, PrOPs provides a report for each patient to aid clinical decision-making, demonstrating the transformative power of digital health technologies in enabling precision medicine and effective patient care pathways.

PrOPs uses a network approach that integrates mutation and transcriptome data in each patient with a knowledge-based genome-wide protein–protein interaction network. Influential ‘key’ driver panels are identified through a specially configured network analysis pipeline. The panels are optimized in size to greedily have the least number of genes while having the highest coverage on the perturbed processes. PrOPs uses two inputs for each patient: (i) transcriptome profile of the tumour sample and (ii) mutation information in the whole exome of the same patient. As a common input for all patients, it also uses (iii) a knowledge-based directed human protein–protein interaction network, (iv) gene function databases, (v) gold-standard gene databases, and (vi) databases containing known actionability information, as well as (vii) known drugs and (viii) the nature of the drug action on the gene products.

As depicted in Fig. 1, the four modules of PrOPs are (i) Module-1, which computes paths of perturbation (*Mutpaths*) for all mutations of the given patient, (ii) Module-2, which identifies *iPanels*, which are shortest panels of driver genes that are upstream of the top-ranked perturbed paths in each tumour sample, (iii) Module-3, which computes PiRS that predicts the effect of the *iPanel* genes on the prognosis of the disease, and (iv) Module-4, which carries out the post-processing of the genes in the *iPanels* to compute the effect of the mutations, an actionability matrix to select ‘actionable’ genes (iAct and iActE) and a recommended individualized ‘Action’. PrOPs identifies actionable driver gene panels, computes a prognosis risk score for each patient, and suggests drugs mapped to the panel genes.

### PrOPs is capable of identification of individual cancer driver gene panels

#### Module-1: *Mutpaths* contain key driver genes

Module-1 has three steps: (i) contextualizing the master knowledge-based human protein–protein interaction network with transcriptome of each patient to generate weighted directed networks, (ii) computation of the shortest paths using the Dijkstra’s algorithm from mutated genes to all other nodes in the network, and (iii) computation of path cost for each path, and ranking in an ascending order and selection of the top 0.01 percentile of paths to generate a subnetwork (*Mutpaths*). The *Mutpaths* thus computed capture the effects of mutations in a given sample in terms of the perceived levels of activities of biological processes. The up- and downregulated genes inferred from the transcriptome, which when integrated with the network perspective, indicate the direction and approximate extent of perturbation of known biochemical pathways, including metabolic, signalling, and regulatory pathways.

**Figure 2.**
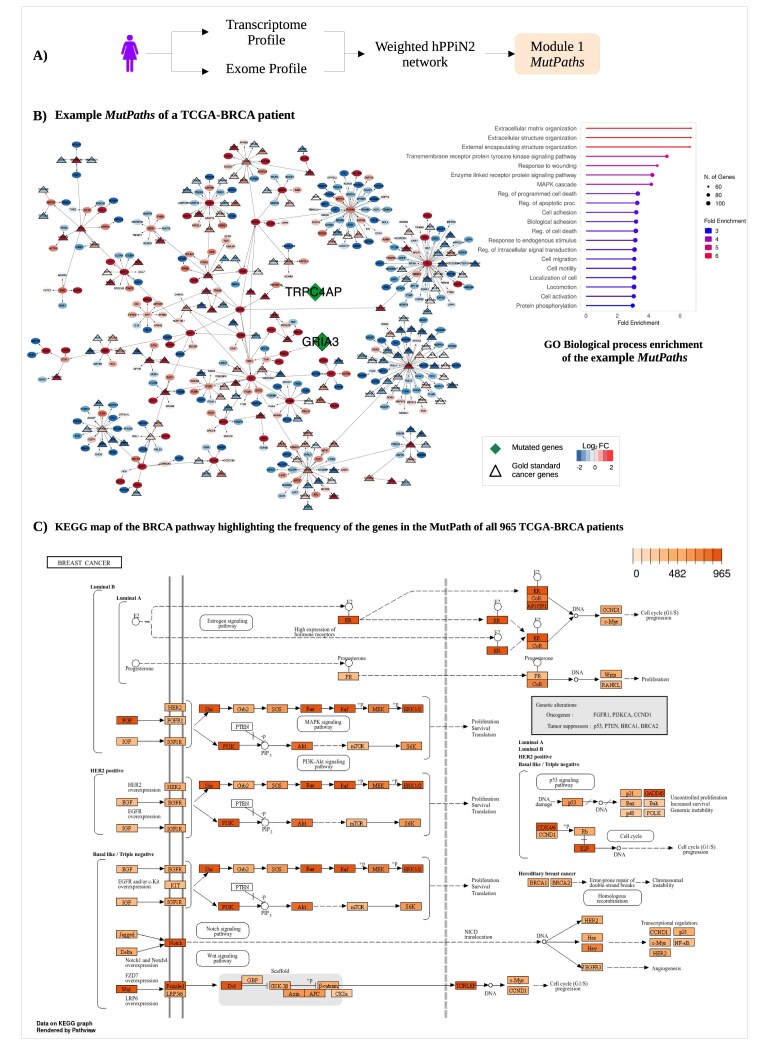
*Mutpaths* captures disease-relevant biological processes. The schematic in panel (**A**) explains the workflow followed to arrive at *Mutpaths*. An example *Mutpaths* of a patient from the TCGA-BRCA cohort is depicted in panel (**B**). The edges are directed based on the flow of information, and nodes are coloured based on their $log_2$ fold change values, while the green nodes depict the mutated nodes in the *Mutpaths*. Gold-standard cancer driver genes in *Mutpaths* are highlighted by triangular nodes with thickened margins. The GO biological process enrichment map (inset) shows that *Mutpaths* represents most of the cancer hallmark pathways. Panel (**C**) shows the KEGG gene map for the Breast Adenocarcinoma pathway. The genes are highlighted based on their frequency of occurrence in the *Mutpaths* of all 965 TCGA-BRCA patients.

The mutation lists from the exome, transcriptome, and phenodata for 6 cancers, summing up to ~2180 samples, were downloaded from the GDC repository database. All non-synonymous mutations, including missense mutations, nonsense mutations, and frame-shift insertions and deletions for the given patient, were included in the mutation list. hPPiN2, a knowledge-based, unbiased genome-wide protein–protein interaction network consisting of 20183 nodes and 255486 interactions, constructed previously in the laboratory, was utilized to construct a transcriptome-weighted (Equations 1 to 3) individualized network for each patient. The first module of PrOPs takes this network as input and computes *Mutpaths* (Fig. 2).

#### Module-2: *iPanels* are concise individualized driver gene combinations

Module-2 uses a greedy maxcover algorithm to compute a minimal set of mutated nodes in each *Mutpaths* such that it covers $>$ 70% of the DEGs in the *Mutpaths*. A rank is assigned to each mutated node based on the number of DEGs in the *Mutpaths* that are reachable within a distance of 4 (reachability within four hops). Nodes are ranked based on the impact they can have in terms of the number of DEGs downstream of that node. Starting from the highest-ranked mutated node, the maxcover algorithm identifies a minimal set of nodes that have an impact on at least 70% of the DEGs in the *Mutpaths*, which are in essence individualized driver mutation panels in each patient (referred to as the *iPanels* hereafter). For each node in an *iPanel*, a Netscore is computed based on Equation (4), which indicates the extent of influence that a given mutated gene has on the *Mutpaths*. Further, a consolidated PrOPs individual Risk Score (PiRS) is computed according to Equation (8) to predict the prognosis of each patient. Using individual *iPanels* of all patients in a given cohort, we also obtain a cohort-level list of driver genes for each cancer. This is achieved using a Condorcet ranking implemented with the votesys library in R. This cohort list is further used to compare the performance of PrOPs with other methods.

##### *iPanels* can be identified in almost all patients

Building on the *Mutpaths*, Module-2 identifies *iPanels* in each patient sample, defined as the shortest panels of driver mutations. PrOPs successfully generated *iPanels* for 2130 of the 2180 samples analysed across the six cancer types. In the remaining samples where no panel was identified, patients exhibited very few mutated or perturbed genes, and no influential mutations were detected in the *Mutpaths* under the default settings. Adjusting the parameters to compute larger *Mutpaths* by expanding the cutoff for perturbed genes from the default top 0.01 percentile to the top 0.02 percentile resolved this issue in 50 samples.

##### *iPanels* are concise

**Figure 3.**
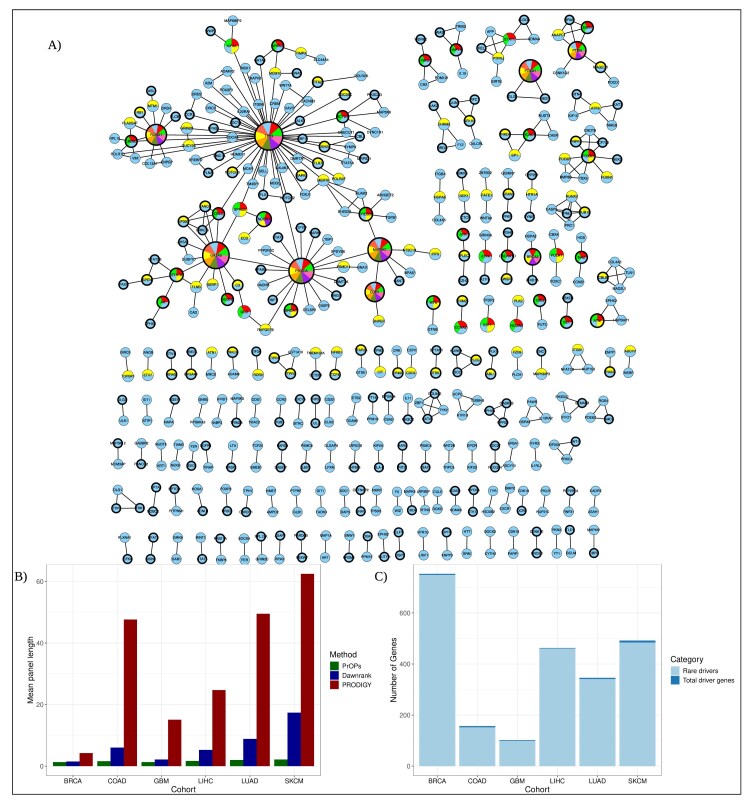
(**A**) *iPanel* gene combinations were unique to each patient, with limited overlap in genes across panels. The connections between *iPanels* from all 965 patients in the TCGA-BRCA cohort are shown. Node size and pie-slice proportions indicate the frequency with which each gene occurs in different *iPanels*. Gold-standard cancer driver gene nodes are highlighted with thickened margins. (**B**) Mean panel sizes obtained from the three methods across all six cohorts were compared; PrOPs *iPanels* were consistently smaller, containing fewer genes per panel. (**C**) In each cohort, PrOPs identified patient-specific driver genes regardless of their mutation frequency, with most *iPanel* genes present in fewer than 2% of patients.

The size of *iPanels* ranged from 1 to 4 in BRCA, 1 to 5 in COAD, 1 to 4 in GBM, 1 to 8 in LIHC, 1 to 7 in LUAD, and 1 to 9 in SKCM, demonstrating that the panels are generally small. Moreover, the *iPanels* were consistently smaller than those identified by DawnRank and PRODIGY for the same individual (Fig. 3B). In 57% (1244/2180) of patients, including 685 patients in BRCA, 78 in COAD, 109 in GBM, 182 in LIHC, 79 in LUAD, and 111 in SKCM, a single gene is sufficient to form an *iPanel* when it is associated with a large number of perturbed downstream genes in the *Mutpaths*.

##### *iPanels* identify well-known drivers as well as rare drivers

We observe that all *iPanels* (size $>$1) are unique as combinations in each patient, although, as can be expected, some genes are seen to occur frequently as part of different panels (Fig. 3A). Nearly half of the genes in the *iPanels* are known to be associated with the respective disease, either broadly or specifically, while several represent newly identified candidate mutations. An overlap analysis with the gold standards showed that ~50% (873 of the total 1869 genes) of the *iPanel* genes of all the patients put together are gold-standard genes. The same phenomenon was observed across all six cancer cohorts—gold-standard genes accounted for 43.4% of *iPanel* genes in BRCA (327/753), 58.5% in COAD (92/157), 50.9% in GBM (52/102), 49.7% in LUAD (172/346), 47.7% in LIHC (221/463), and 53.6% in SKCM (264/492). Details of *iPanel* genes in all cohorts are provided in Supplementary Table S1.

The effect the panels have on the *Mutpaths* is seen to be biologically significant, as *Mutpaths* contain functional perturbations in different patients. For example, an *iPanel* of size 3 encompassing genes FOXJ1, TP53, and MERTK and the corresponding *Mutpaths* is seen to cover key perturbed processes such as cell cycle checkpoint regulation and PLK1 signalling pathway in breast cancer. BRCA1/2, a known mutation in breast cancer, was identified in *iPanels* of 9 patients and is known to be associated with genes involved in the TGF-$\beta$ pathway, eventually leading to a disrupted cell cycle regulation. *iPanel* genes that are present in < 2% of the cohort are classified as rare driver genes. These rare genes comprise the majority of the *iPanels*: BRCA: 749 genes out of the total 753 *iPanel* genes, COAD - 152/157, GBM - 99/102, LIHC - 461/463, LUAD - 341/346, and SKCM - 484/492 (Fig. 3C).

In the TCGA cohort, the four distinct subtypes of melanoma (TCGA-SKCM), BRAF, NRAS, triple wild type, and NF1 put together, constitute only 25% of the samples, leaving ~75% with no clear mutation association. Similarly, breast cancer subtypes (BRCA1 and BRCA2) account for only $\sim$2.5% (only 24/965 samples in TCGA-BRCA), clearly indicating that other mutations are important. In contrast, PrOPs is able to predict driver gene panels in all of them, enabling personalized descriptions of the driver genes and precision guidance of actionability.

### Performance benchmarking of PrOPs

**Figure 4.**
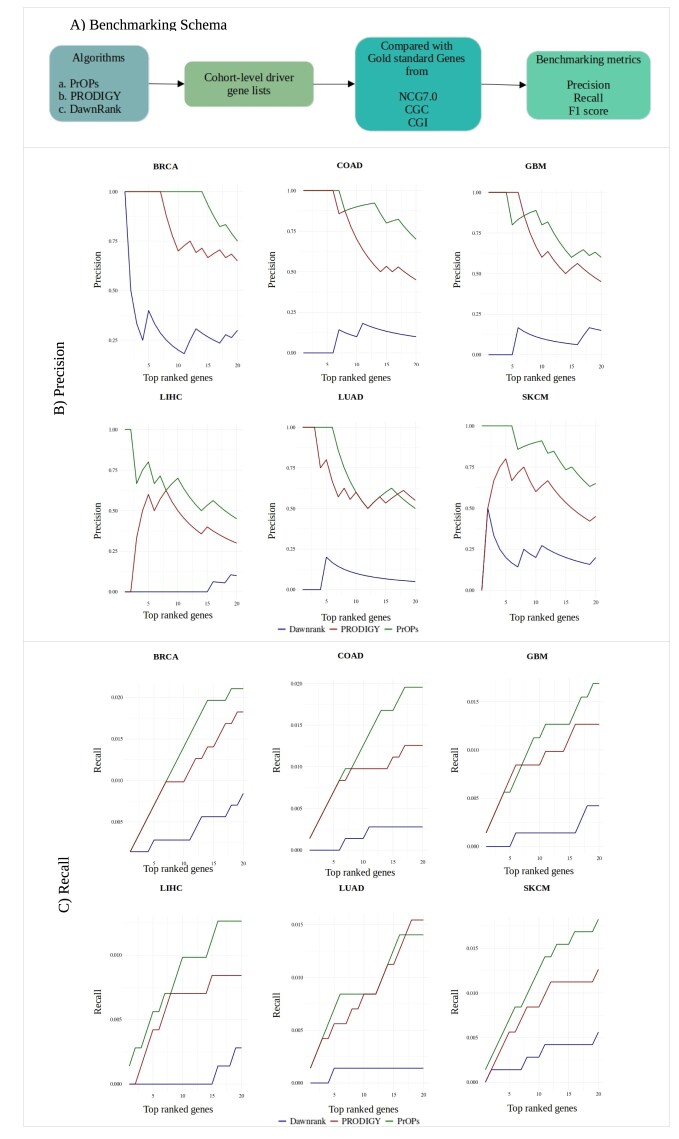
Benchmarking schematic followed is described in (**A**). Cancer driver genes from NCG6.0, CGC, and CGI were consolidated and used as the reference gold-standard list. The top 20 genes from all three methods were benchmarked against the gold-standard cancer driver genes. The precision and recall plots in panels (**B**) and (**C**), respectively, show that our method performed better than the others.

The performance of PrOPs was compared with driver genes identified by DawnRank and PRODIGY, which are single-sample, network-based methods. To compare performance across different cohorts across the three methods, we computed Condorcet ranking using votesys library [30]. Briefly, the PrOPs driver lists for individual patients are used as an input into the Condorcet ranking, which outputs a cohort-level ranked list for each cancer type. The same is carried out for all three methods: PrOPs, PRODIGY, and DawnRank. The lists obtained are in a comparable framework and hence used for benchmarking against the gold-standard driver gene list (Supplementary Table S2) by testing how many of the top 20 ranked genes were gold-standard genes, or, in other words, known driver genes of that particular cancer. The results are shown in precision and recall plots (Fig. 4), which clearly indicate the significantly superior performance of PrOPs as compared to the other two methods.

### Module-3: prognostic potential of driver combinations

The driver mutations identified by PrOPs in each cancer cohort were analysed for their effect on the prognosis of the disease. A univariate Cox proportional hazard model was constructed to estimate the HR of individual driver genes in the cohort. The cohort-level lists obtained through Condorcet ranking and used for benchmarking also served as the basis for this analysis. HR is computed based on the survival information in the population and indicates the survival risk potential of each gene. An HR value $<\!1$ indicates low risk, while a value $>\!1$ indicates high risk of survival. The cohort-level lists obtained through Condorcet ranking and used for benchmarking also served as the basis for this analysis. One of the main advantages of PrOPs is that it provides patient-level driver gene lists, which we use to obtain patient-level insights by computing a PiRS for each patient. Briefly, PiRS captures how many high HR driver mutations are present in a given patient (*iPanel*) and thereby provides a combined risk profile for that patient. PiRS was formulated for each patient using Equation (8), which has the net score and HR for each gene in the *iPanel* as its components, where the net score gives the connectivity of the driver gene to the perturbed genes in the top response network, while the HR accounts for the effect on prognosis.

**Figure 5.**
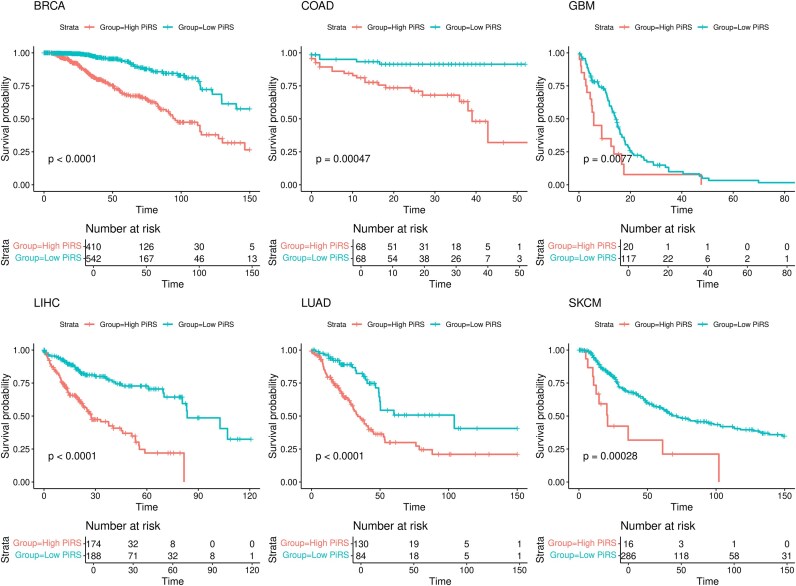
PrOPs risk score is capable of predicting the prognosis of the disease. The patients are grouped into low PiRS and high PiRS groups based on a screening exercise to identify optimal prognostic stratification (Supplementary Fig. S1). The Kaplan–Meier plots show the survival difference between the high-PiRS and low-PiRS groups in all the cohorts. The low-PiRS group has significantly better survival compared to the high PiRS group.

PiRS was able to predict survival differences among the patients. We did a systematic scanning of the PiRS values in each cohort to select a cutoff score that can significantly explain the survival differences in the PiRS-high and PiRS-low subgroups of patients. The details are further explained in Supplementary document section 1. Figure 5 represents the survival differences between the PiRS-low and PiRS-high groups. From our analysis, we found that patients in the PiRS-high group had a poor prognosis compared to those with low scores. We have also discussed the relation of the score with stages and disease progression in each cohort in the Supplementary document section 2 along with the Supplementary Fig. S2. Because of the NetScore component, PiRS was still able to explain the prognostic effect of the gene across stages and disease duration among patients sharing the same *iPanel* gene. A case study of BRCA cohort patients with TP53 as the *iPanel* gene is presented in the Supplementary Fig. S3.

### Module-4: actionability

Next, we checked for the potential clinical actionability of all the driver genes identified by PrOPs. We tested the extent to which *iPanel* genes were mapped to approved drugs, as such mapping could inform the selection of treatment options for individual patients. To compile the reference set of putatively actionable genes, we consulted multiple sources: (i) the COSMIC actionability database, which provides DMD associations; (ii) DGIdb, a drug–gene interaction database encompassing the known and potential druggable genome, with drugs mapped to each gene and cross-referenced with OncoKB, PharmGKB, Drug Target Commons, and JAX OncoCore; (iii) TARGET, which catalogues genes with potential therapeutic, prognostic, or diagnostic implications in cancer patients; and (iv) the Therapeutic Target Database (T2D). Collectively, these databases provide gene-to-drug mapping. In addition, we incorporated gene family information for potentially druggable genes from the Cancer Druggable Gene Atlas (TCDA) and functional annotation (oncogenes or tumour suppressor genes) from OncoKb. Gene lists with approved targets were retrieved from the DGIdb (last accessed in December 2023). Put together, 1068 genes were identified as actionable across these resources, with 2176 drugs mapped to them. Supplementary Table S3 provides the master actionability database used in this analysis. The *iPanel* genes were queried against the reference set, and the corresponding drug information was retrieved and summarized into a report for each patient.

**Figure 6.**
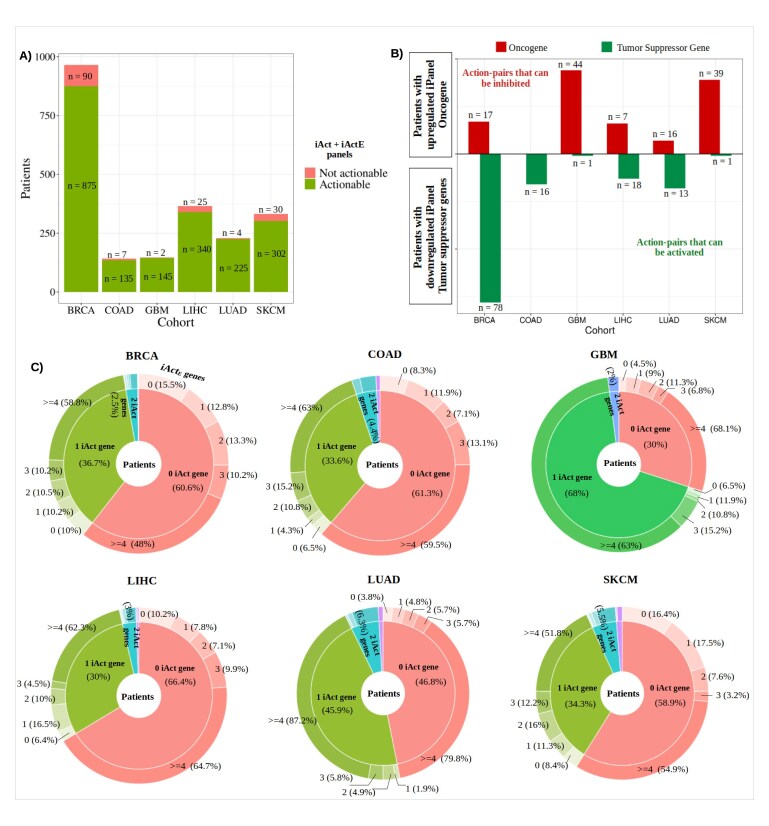
Actionability status of the *iPanel* genes. (**A**) The stacked barplot explains the number of patients with at least one actionable gene based on their iAct and iActE panels. (**B**) The action-pair statistics for each cohort are calculated based on the functional status (oncogene or tumour suppressor gene) and gene expression (up- or downregulated) of the *iPanel* genes. The red bars represent the patients who have upregulated oncogenes that could potentially be inhibited, and the green bars represent patients with downregulated tumour suppressor genes that could be activated. (**C**) The pie–doughnut chart gives the breakdown of the patient distribution of iAct and iActE panels in each cohort. The inner pie represents the distribution of the patients for the iAct panels, and the outer pie represents that of the iActE panels.

We observe that in individual patients, 17.97% (336/1869) of all *iPanel* genes are actionable, and 41.23% (899/2180) of patients contain at least one actionable gene in their *iPanels* (inner pie in the Fig. 6C). At the cohort level, we observe the following statistics for patients who have one or more actionable genes in their *iPanel *(cohort—actionable patients/total patients): BRCA - 376/965, COAD - 53/142, GBM - 103/147, LIHC - 122/365, LUAD - 118/229, and SKCM - 127/332. These actionable iPanel genes constitute the iAct genes, the *iPanel* genes that could be acted upon.

Next, we sought to prune the list of actionable *iPanel* genes based on the expression values of the individual *iPanel* genes in that patient and triaged only those that have a perturbation in disease with a fold change of $>\!2$. The nature of the recommended ‘action’ takes into account the following: (i) actionability of the genes, (ii) gene expression value and whether it is up- or downregulated as compared to a control group, (iii) the functional role of the gene- whether it is an oncogene or a tumour suppressor gene (as given in OncoKb), and (iv) the nature of the available drug for the target—whether it is an inhibitor/antagonist or an activator/agonist. This results in the mapping of the targetable gene to the known drug called the ‘action pair’. This provides us with a small set of perturbed *iPanel* genes that are actionable (Fig. 6B). Most upregulated oncogenes have inhibitor drugs, while the perturbed tumour suppressor genes (up- and downregulated) have approved activator drugs. The iAct genes are classified under two different axes: (i) up- or downregulated and (ii) oncogenes or tumour suppressors. As described in Module-4 of the section ‘Module-4: Actionability’ and Fig. 6B, the actionability of upregulated oncogenes is paired with inhibitors, while the actionability of tumour suppressors (up- or downregulation) is paired with agonists. The pairing optimizes inhibition of tumour growth either through direct suppression of growth-linked genes or indirectly by enhancing the action of natural tumour suppressors through available agonists.

Further, among the *iPanel* genes, we observed some to be clearly important for the pathology, but with no approved drugs mapped to them. To cater to such scenarios, we selected perturbed downstream genes of all the *iPanel* genes to check for their actionability status. We observed TP53 to be the most frequently perturbed tumour suppressor gene, present in 16.05% (350 out of the total 2180 patients) within the *iPanel* genes. TP53 is also included in many cancer gene panel tests and is a gold-standard cancer driver gene. Although DGIdb categorizes TP53 as targetable based on clinical trials, TP53 is still largely considered “undruggable” with no approved drugs to date. In addition to TP53, a significant portion of the actionable *iPanel* genes belong to the Transcription Factor (TF) family. We therefore extended the *iPanel* genes to their corresponding DEGs, representing their closest network neighbours. As in the greedy cover algorithm (Section ‘Module-2: *iPanels* are concise individualized driver gene combinations’), DEGs up to the fourth neighbour of the mutated *iPanel* genes in the Module-2 networks were considered, and the actionability analysis was repeated. We term these as iActE (iAct extended) panels. We found that for most of the patients lacking a targetable *iPanel* gene, the corresponding downstream perturbed genes were targetable. Through this analysis, 93% of the samples were found to have at least one gene that could be targeted in their iAct and iActE panels (Fig. 6A).

Supplementary Table S4 gives the breakdown of the number of individual *iPanels* that are actionable based on their expression profiles and functional annotation, along with the names of the drugs and the mode of action of the drug given in DGIdb. Some examples of frequently identified ‘actions’ from our algorithm are Carfilzomib, Bortezomib, and Dacomitinib, which are proteasome inhibitors that are commonly used in the clinic to treat various cancers. Based on this, PrOPs generates a recommendation report for each patient to aid the clinical decision-making process.

## Discussion

Over the past decade, it has been well-recognized that precision oncology is the key to improving the success rates of cancer treatment and enhancing patient outcomes, with next-generation sequencing playing a central role in it. For its implementation in a clinical setting, however, several barriers must be crossed, the most critical being the analysis of the NGS-derived omics data to identify a concise set of key actionable alterations and associated ‘actions’ from the tumour. The currently available methods identify most frequently observed mutations, typically as part of large mutation panels. However, given the high levels of complexity and heterogeneity that tumours present, translating such large mutation panels into clinical ‘action’ recommendations is highly challenging and typically works in a small subset of patients with signature mutations. The digital health framework PrOPs that we present here addresses several of these challenges and provides a clinically deployable solution for precision oncology. The key advantage of PrOPs is that it identifies short actionable mutation panels in each individual patient, which are central to the perturbations present in their molecular networks. PrOPs also reports a risk score for each patient, which indicates their survival prognosis. It also makes use of known target-drug associations and identifies approved drugs for each patient. A limitation of the study is that only known associations are predicted as action pairs, and the approach also leaves out the variant-specific resistance to drugs. These can be overcome by including an additional filter based on drug resistance mechanism and the possible presence of resistance-inferring mutations in the other genes in that patient.

The core algorithm in PrOPs was rigorously benchmarked against DawnRank and PRODIGY, two recently published methods that use similar information to identify personalized driver genes, and demonstrated higher sensitivity and specificity. Another advantage of the PrOPs is its use of the network-mining algorithm in the *Mutpaths* module, which identifies mutations with downstream cascading effects that have previously been linked to pathology-relevant alterations. The network used as an input into *Mutpaths* is a knowledge-based genome-wide protein–protein interaction network that integrates both structural interactions and functional influences, facilitating an unbiased search for alterations in each patient sample relative to a suitable control. Mutations in the top-perturbed network are linked to the largest set of expression-altered genes, connected functionally among themselves, facilitating the shortlisting of pathology-relevant mutations in the first module and the final selection of *iPanel* genes in the second module. Our approach can be envisaged to have additional benefits. Knowing the target in an individual patient will help in precision target identification and tailoring therapy, thus reducing the chances of therapy resistance and thereby treatment failure.

A risk score PiRS is computed by PrOPs for each sample based on their *iPanel* genes. A clear correlation of the PiRS was evident with the living or deceased status of the patients and across the clinical parameters, such as the survival months and stages of the disease, indicating the clinical relevance of the identified panels. PiRS scores enable precise and individualized risk stratification, which goes beyond conventional factors. It helps identify high-risk individuals, who can benefit from proactive management of tests and interventions as necessary, for example, more frequent screening or evaluations or more aggressive therapy.

For actionability prediction of the *iPanel* genes, each gene in the panels is tagged with expression and functional information, allowing the pairing of up-regulated oncogenes with their known inhibitors and tumour suppressors, whether up- or downregulated, with their agonists. The pairing aims to bring out ‘inhibition of tumour growth’ either through direct suppression of growth-linked genes or indirectly by enhancing the action of natural tumour suppressors through available agonists. The limitations of current action-pair predictions are that they are restricted to known approved drugs and are dependent on the drug-target mappings provided in literature and databases. It does not take into account any off-target effects that may be present, nor does it take into account if the mutated target in the given individual has the ability to bind the mapped drug in exactly the same way as the reference protein for which drug binding would be characterized. We believe our work provides a rational framework to make such predictions that are testable in the field through suitably planned clinical studies, from which changes to patient outcomes can be evaluated.

## Supplementary Material

lqaf177_Supplemental_Files

## Data Availability

The source code of the PrOPs algorithm is deposited in the GitHub repository at https://github.com/chandralab-iisc/PrOPs.git and on Zenodo repository at the following link https://doi.org/10.5281/zenodo.17279026
