# Supplementary material for: A new algorithm Precision OncoPanels (PrOPs) identifies short individualized actionable panels that can guide cancer treatment: a pan-cancer analysis of TCGA cohorts
Source: NAR Genom Bioinform. 2025 Dec 8;7(4):lqaf177. doi: 10.1093/nargab/lqaf177 (PMC12684385; doi:10.1093/nargab/lqaf177)
Supplement: lqaf177_Supplemental_Files [file lqaf177_supplemental_files.zip › Supplementary_document_1.pdf]

PiRS and survival analysis across cancer cohort:

This is the supplementary figure for the main Figure 5. The PiRS score calculated for all patients was screened to arrive at a cutoff threshold to categorise patients into two (low and high PiRS) groups.

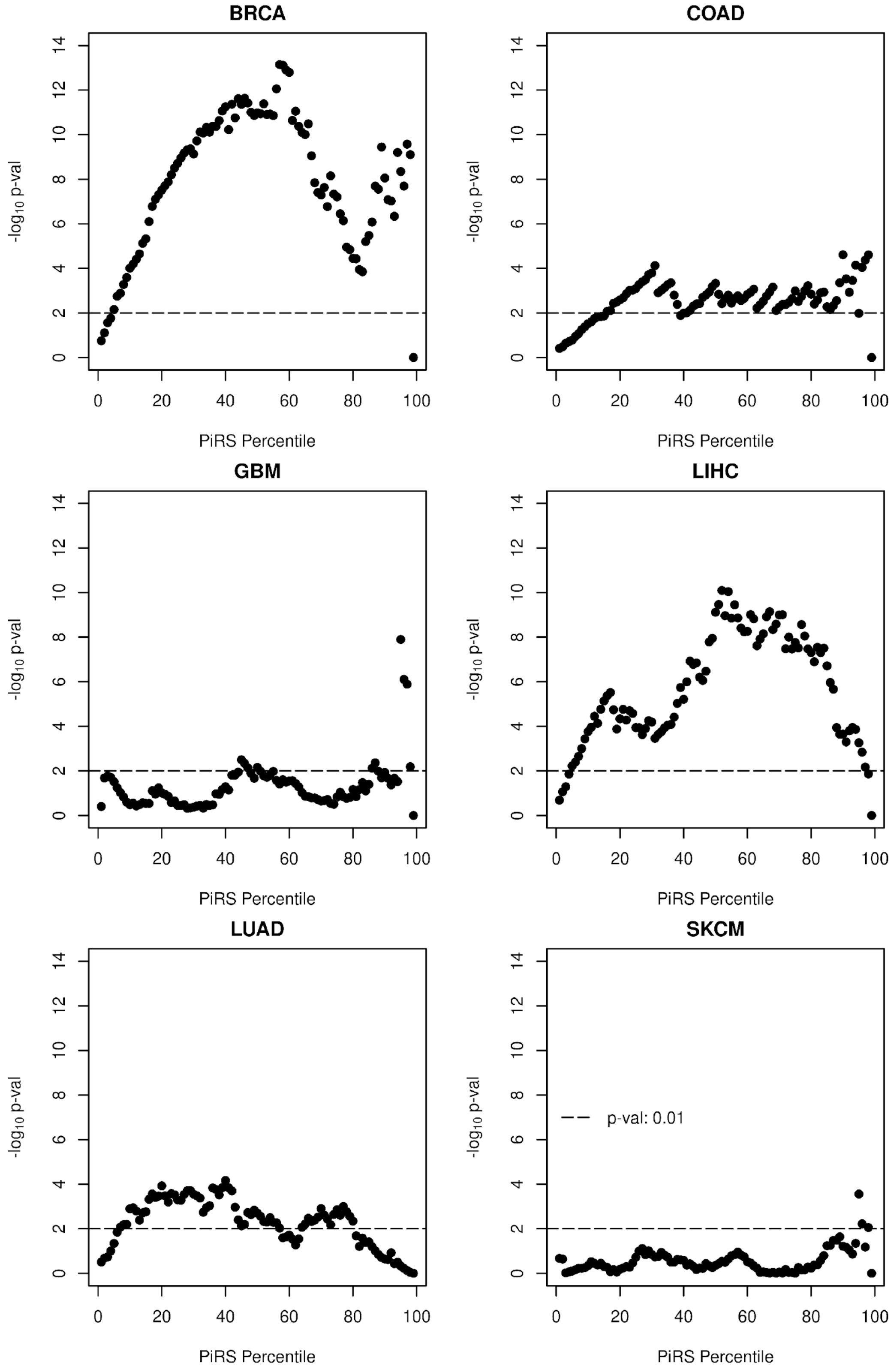

Supplementary figure 1: The PiRS scores are plotted on the x-axis as their percentile. Survival analysis was carried out for each percentile and the resulting p-value from the survival model is plotted in negative $\log_{10}$ scale in the y-axis. The horizontal line refers to p value < 0.01 indicating the significance. The scores above this line were considered for categorising the patients.

The cutoffs were chosen based on the minimum p-value that also happened to be around the 50th PiRS quantile. For COAD, GBM and SKCM, we empirically chose the scores to get a nearly equal proportion of patients between groups with $p < 0.01$ significance. The cut-off scores selected are given in the following table along with the number of patients in each group.

Figure 5 in the main document shows the Kaplan-Meier plots to understand the difference in survival between the groups. Patients in the low PiRS had better survival than those in the high PiRS group.

**Cohort**	**PiRS cutoff**	**Number of patients in PiRS-high group**	**Number of patients in PiRS-low group**
BRCA	8.54	410	542
COAD	15.91	68	68
GBM	62.11	20	117
LIHC	21.84	174	188
LUAD	23.41	130	84
SKCM	162.18	16	286

Supplementary method:
1. Node weights were calculated independently for each sample. In addition, a cohort-level analysis was also carried out by considering different patient samples as biological replicates (pool of tumour samples versus pool of non-tumour control) in the cohort. The resulting MutPaths showed similar enrichment patterns as the individual patient-specific MutPaths.

2. **Correlation of the risk score (PiRS) with the clinical parameters**:
We are able to see differences in the resulting iPanels obtained across the stages of all 6 cancer types which includes several well-classified subtypes in each. The survival analysis and subsequent PiRS calculation for individual patients based on their iPanels show an increasing score trend with increasing stages. Supplementary figure 2 depicts the differentiating trend of the PiRS score between Living and Deceased patients across cancer stages.

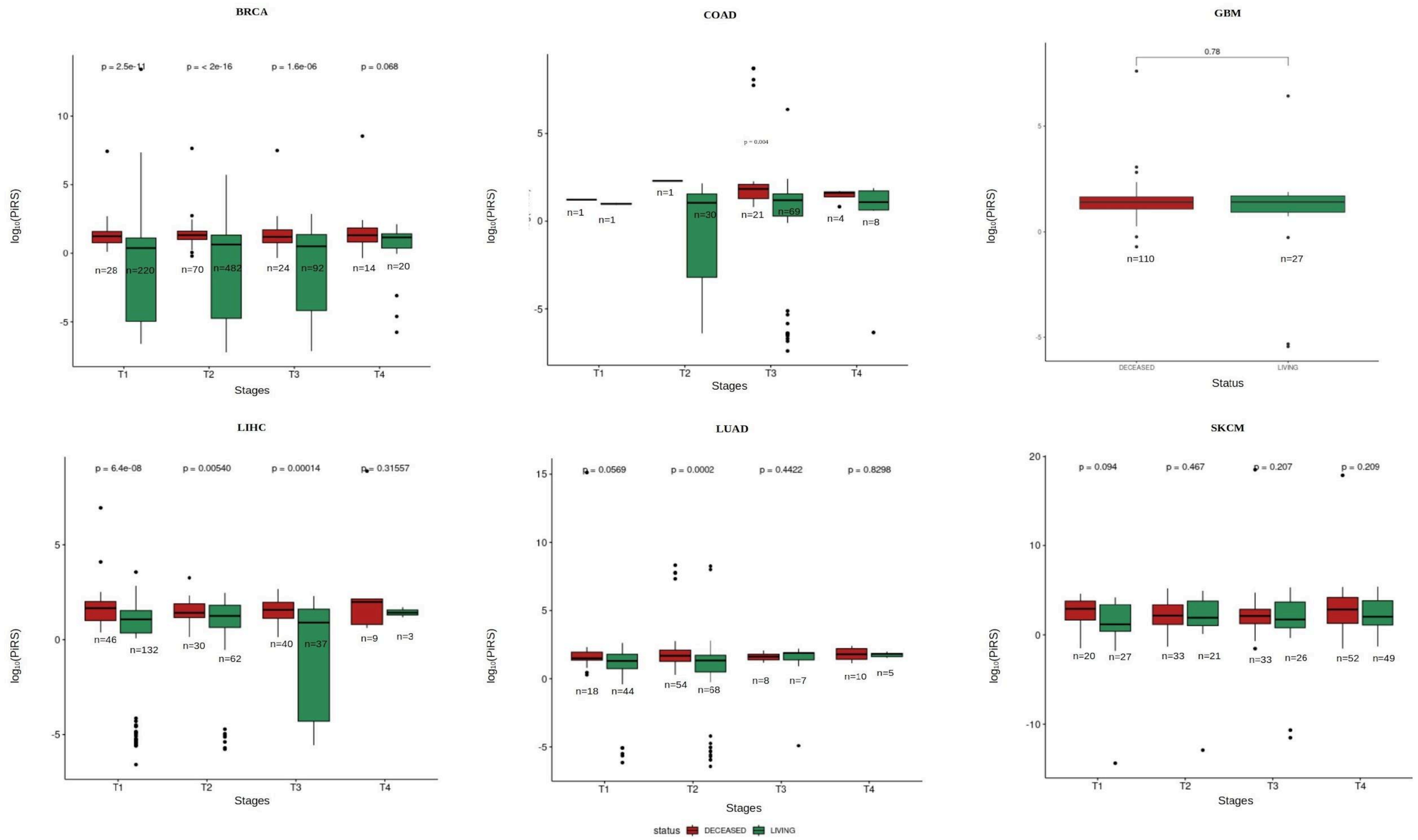

Supplementary figure 2: Differences in the PiRS scores among the Living (green) and Deceased (red) patients across cancer stages in each cohort

Moreover, the NetScore component of the PiRS scoring scheme explains the prognostic effect of the identified iPanel genes with respect to the patient's clinical parameters such as the cancer stage, disease duration, and status at the end of the study.
A case study of 6 patients from TCGA-BRCA presented in supplementary figure 3 proves that PiRS is capable of explaining the effect of the driver gene panel on the clinical phenotype. We observe the association of the score with the overall survival months, cancer stages and status of the patient. In scenario 1, the patient P2 with Deceased status have significantly higher PiRS despite having similar survival months as of P1. In scenario 2, though the survival months were the same for P3 and P4, PiRS is higher for the stage 4 patient (P4). In scenario 3, P6 have higher PiRS corresponding to the higher survival month and the cancer stage.

		Overall Survival months	PiRS	Status
Scenario1	P1	19.9	9.06	Alive
	P2	24.7	154.4	Deceased
Scenario2	P3	17.21	18.6	T1
	P4	18.76	81.06	T4
Scenario3	P5	74.87	5.07	T1
	P6	139.06	26.3	T4

Supplementary Figure 3: Comparison of the PiRS (PrOPs individual Risk Score) of 6 patients from the TCGA-BRCA cohort with the same driver gene panel, TP53. Due to the differences in the network topology of the MutPaths and their downstream connectivity to the perturbed genes, TP53 has different NetScores in all 6 patients. This contributed to the different PiRS in the patients despite them having the same panel gene.

3. **Benchmarking**:

We carried out benchmarking with 2 methods - DawnRank and PRODIGY. DawnRank was benchmarked against DriverNet, PARADIGM-Shift, CHASM and Oncodrive-FM and PRODIGY against DawnRank and SCS, while SCS was benchmarked against DawnRank and OncoIMPACT. Figures shown here, taken from their respective papers clearly indicate DawnRank and PRODIGY to be comprehensive best-performing methods to identify driver mutations. Hence, we chose to benchmark against these 2 methods.

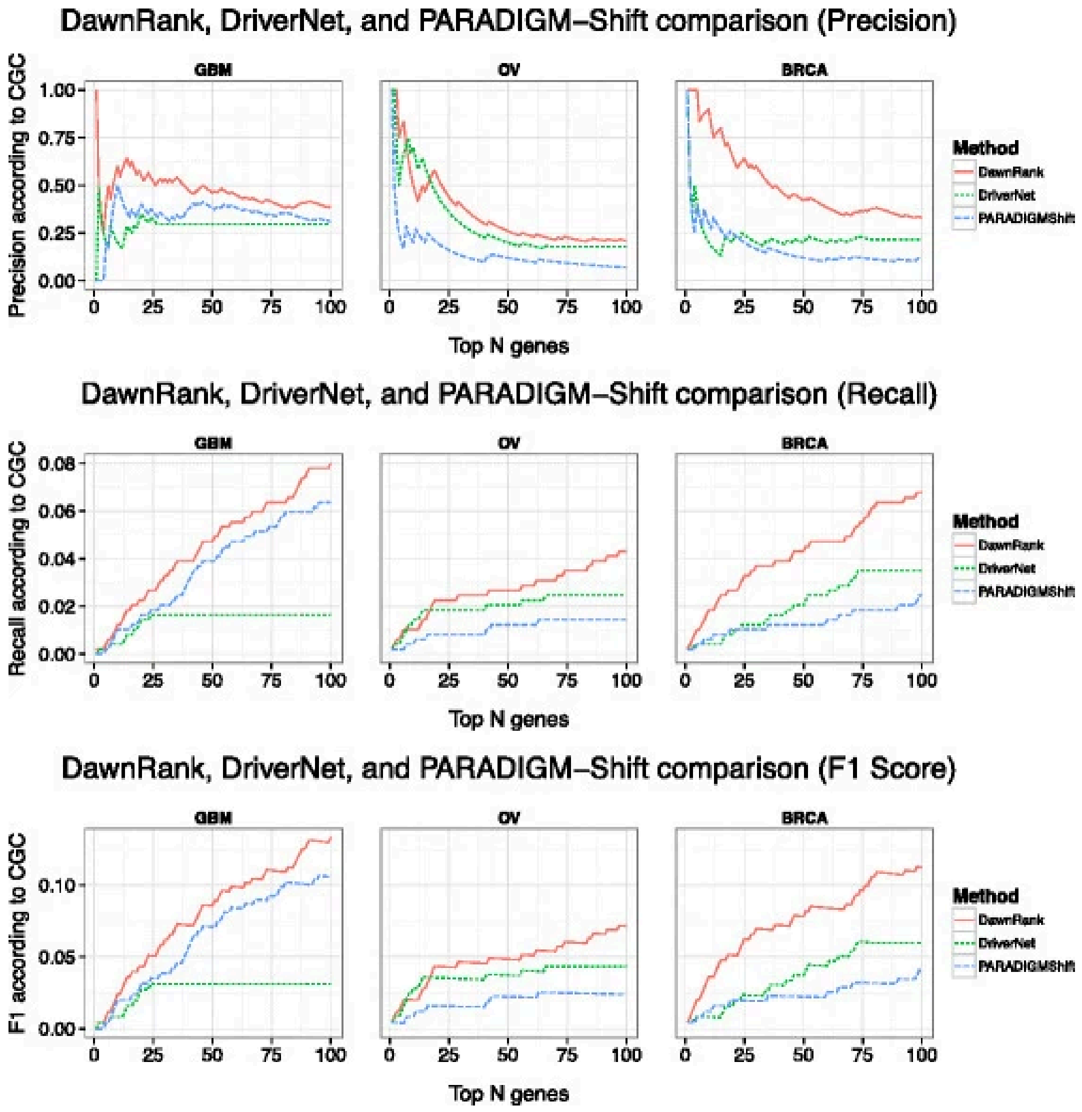

This figure taken from the paper *DawnRank: discovering personalized driver genes in cancer,* describes the comparison of DawnRank with DriverNet and PARADIGM-Shift. All the measures, namely Precision, Recall and F1-score show that DawnRank performance is superior.

(Reference: Hou, J. P. & Ma, J. Dawnrank: discovering personalized driver genes in cancer. Genome Medicine,6 (2014).)

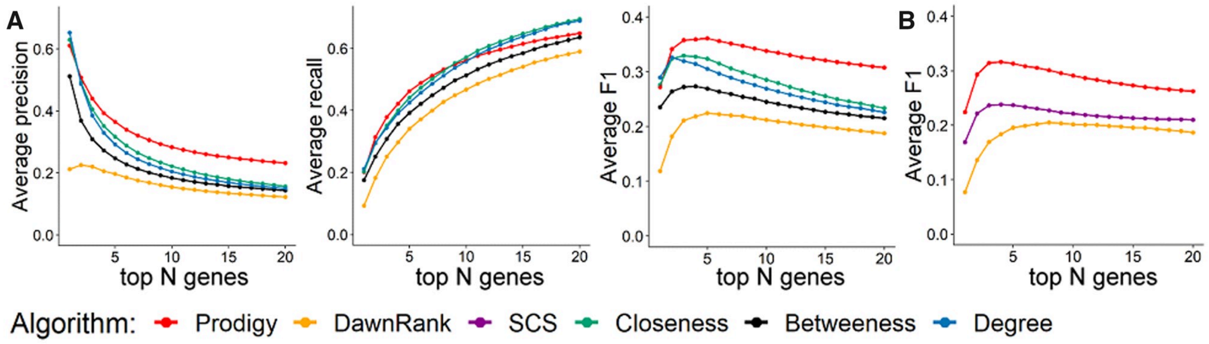

Similarly, the above figure 5 from *Prodigy: personalized prioritization of driver genes* depicts the better performance of PRODIGY as compared to DawnRank, SCS and network centrality measures like closeness, betweeness and degree centralities.

(*Reference: Dinstag, G. & Shamir, R. Prodigy: personalized prioritization of driver genes. Bioinformatics 36, 1831–1839 (2019).*)

Overall, our algorithm PrOPs has been directly compared with 2 and indirectly with a total of 6 different algorithms. This makes the benchmarking quite robust. We have explained the working principle of each algorithm in the table below.

**Method**	**Working principle**	**Input**	**Output**
PrOPs	Uses max cover greedy algorithm to identify driver mutations related to the perturbed genes in the protein-protein interaction network	Patient-specific mutation and gene expression list	Patient-level; Personalised panel of ranked driver mutations and their corresponding prognostic risk score and actionability status; typically concise panels
Dawnrank	Uses the random walk approach of PageRank to identify mutations that have higher connectivity in the protein-protein interaction network.	Patient-specific mutation and gene expression list	Patient-level; List of ranked patient-specific driver genes; typically concise panels
DriverNet	Uses the network topology of the transcriptional regulatory network to identify driver mutations	Mutation and gene expression list	Cohort-level list of driver genes.
PARADIGM-Shift	Belief-propagation algorithm that infers gene activity based on CNV and gene expressions in specific biological pathways	Copy number variation, Methylation status, Gene expression and specific biological pathways	Cohort-level; Predicts the functional status of the driver genes as either loss-of-function or gain-of-function mutations.
CHASM	Random forest classifier trained on 49 features to distinguish the driver mutations from synthetically generated passenger mutation list.	List of cancer-specific mutated genes	Cohort-level; List of cancer-specific driver genes
OncodriveFM	Computes the functional impact of each variant in the cohort and the functional bias of those variants to occur in specific genes and pathways	List of cancer-specific single-nucleoti de variants	Cohort-level; List of FM-biased driver genes and pathways
PRODIGY	Uses the prize-collecting Steiner tree model to rank the driver mutations based on their aggregated impact on the dysregulated pathways	Patient-specific mutation and gene expression list<br>List of genes associated with	Patient-level; Patient-specific ranked list of driver genes (panel are typically large, 10-40+ genes)

		REACTOME pathways.	
SCS	Identifies driver genes based on their impact on the gene expression profile in the disease state compared to a normal state.	Patient-specific mutation and gene expression list	Patient-level; Ranked list of patient-specific driver genes.
OncoIMPACT	Identifies driver genes that are associated with the dysregulated network modules	List of cancer-specific mutations and gene expression profiles	Patient-level; Patient-specific driver gene list.
