# Supplementary material for: A new algorithm Precision OncoPanels (PrOPs) identifies short individualized actionable panels that can guide cancer treatment: a pan-cancer analysis of TCGA cohorts
Source: NAR Genom Bioinform. 2025 Dec 8;7(4):lqaf177. doi: 10.1093/nargab/lqaf177 (PMC12684385; doi:10.1093/nargab/lqaf177)
Supplement: lqaf177_Supplemental_Files [file lqaf177_supplemental_files.zip › Supplementary_document_2.pdf]

This short tutorial demonstrates the usage of PrOPs for obtaining the iPanel for an example patient, using data for one glioblastoma (GBM) patient from TCGA.

Patient ID: TCGA_06_0157
The input files are present in the tutorial_data/ folder in the Github repository - https://github.com/chandralab-iisc/PrOPs

Step 1: Preparation of the input files

The input files in this case had their source in the raw transcriptomic and mutation data downloaded from TCGA via the GDC client. Differential gene expression analysis was carried out in EdgeR to get the fold change values of the patient with respect to the non-tumour samples in the cohort.
PrOPS requires three input files - namely the node weight file, edge weight file and the network file. The format of each file is illustrated with examples that display the initial rows.

- Node weight file (TCGA_06_0157_01A_nodewt.tsv), containing genes and their fold changes values with respect to all non-tumour samples in the cohort. An example of the file format is given below.

A1BG	0.173105236794845
A1BG-AS1	0.0944457169485346
A1CF	0.388810461125045
A2M	3.45634289075861
A2M-AS1	0.53452444357028
A2ML1	3.18553346470023
A3GALT2	0.930505876456868
A4GALT	0.776709962040854
AAAS	1.98999613771824

- Mutation file (TCGA_06_0157_01A_mutated_genes.txt), containing the list of non-synonymous mutations in the patient. An example of the file format is given below.

SPTA1
SERPINC1
SYT2
HSD11B1
RYR2
SNRNP200
TBR1
TTN
PDE1A

- Network file (hPPiN2.tsv), the curated in-house human protein-protein interaction network. An example of the file format is given below.

Source	Target
A1BG	ANXA7
A1BG	CDKN1A
A1BG	TK1
A1CF	SYNCRIP
A2M	ANXA6
A2M	ANXA7
A2M	ATF7IP
A2M	C11orf58

- The DEG file (TCGA_A6_0157_01A_deg.txt) contains the list of genes differentially expressed with respect to all non-tumour samples in the cohort. (more than two-fold upregulation/downregulation with p-value < 0.05). An example of the file format is given below.

A1BG
A1BG-AS1
A2M
A2ML1
AACS
AAK1
AAR2
AARD
AATK

Step 2: Constructing the patient-specific network by calculating the edge weights by contextualising the hPPiN2 network with the fold change values of the sample.

Code used: gawk -v nodefile= TCGA_A6_0157_01A_nodewt.tsv -v mode="Active" -f edge_wt_calc.awk hPPiN2.tsv > TCGA_A6_0157_01A_edgewt.tsv

This step calculates the edge weights. A patient-specific network is constructed based on the genes present in that sample and the interaction information from the hPPiN2 network. The edges in the patient network are made context-specific by incorporating the fold changes of the node pairs in the edge using the eq (2). An example of the output file is given below.

A1BG	ANXA7	3.33042301487761
A1BG	CDKN1A	1.2578289556994
A1BG	TK1	0.742679328903975
A1CF	SYNCRIP	1.21371949054829
A2M	ANXA6	0.556175353477814
A2M	ANXA7	0.745325691931386
A2M	ATF7IP	0.436069800592723
A2M	C11orf58	0.532187875611596
A2M	CDK2AP2	0.469262337562442
A2M	CDKN1A	0.281493441688951
A2M	CELA1	0.0872038243572318
A2M	CTSB	0.450388271270053
A2M	FGF2	0.607914619804186
A2M	HMOX2	0.907000976462421
A2M	LRP1	0.46878989232904
A2M	NUDT21	0.648985968273345
A2M	PDGFB	0.54411259450402
A2M	PROC	0.570448899677012
A2M	RAP1B	0.299494417771937
A2M	RPP14	0.6953944727651
A2M	SHBG	0.592455663826372
A2M	SMN1	0.42620023678985
A2M	TGFB2	0.341116628790019
A2M	TGFBI	0.108623282921088
A2M	TGIF1	0.206784189181455
A2M	TK1	0.166206509571227
A2M	TNF	0.722430025702909
A2M	TNFRSF14	0.431929941087859
A2M	TSC22D1	0.581638198018139
A2M	UFD1	0.436064483747784
A3GALT2	B3GALNT1	1.29458836453366
A4GALT	HEXA	0.942456689962111
A4GALT	HEXB	0.840714425543224

Step 3: Generation of all shortest paths containing mutated genes.

Code used: python3 props_sp.py TCGA_A6_0157_01A_edgewt.tsv TCGA_06_0157_01A_mutated_genes.txt 4
Output: TCGA_A6_0157_01A_sp.tsv
Subsequent step: cut -f4 TCGA_A6_0157_01A_sp.txt|sed 1d|sort -n|uniq > TCGA_A6_0157_01A_sp_score.txt;

This step calculates all possible shortest paths from the mutated nodes to all other nodes in the given patient-specific network. The first column represents the source-target node pairs, followed by the pathscore as calculated using the eq (3). The normalised path score is calculated by dividing the pathscore with the path length (number of edges present in the path). An example of the output file is given below.

NodePairs	PathScore	PathLength	NormalizedPathScore	Paths
SERPINC1_KLK6	6.1056275421442	10	0.61056275421442	SERPINC1,LRP1,MDK,STAT1,EN1,HOXA4,HOXA9,TFAP2A,YBX1,KLK6
SERPINC1_PLG	1.25488804713104	6	0.209148007855174	SERPINC1,LRP1,MDK,STAT1,MMP9,PLG
SERPINC1_AKAP1	1.04292513415444	9	0.115880570461604	SERPINC1,LRP1,MDK,STAT1,TBX5,CDKN2A,E2F2,GATA3,AKAP1
SERPINC1_ANAPC10	1.05184155939483	11	0.0956219599449845	SERPINC1,LRP1,MDK,STAT1,TBX5,CDKN2A,E2F2,GATA3,MYBL2,CDK1,ANAPC10
SERPINC1_APBA2	1.74826904263681	10	0.174826904263681	SERPINC1,LRP1,MDK,STAT1,EN1,HOXA4,HOXA9,TFAP2A,YBX1,APBA2
SERPINC1_APBB1	1.02782418605743	9	0.114202687339714	SERPINC1,LRP1,MDK,STAT1,EN1,HOXA4,HOXB7,EGFR,APBB1
SERPINC1_APP	1.03126973053648	9	0.114585525615164	SERPINC1,LRP1,MDK,STAT1,EN1,HOXA4,HOXA9,TFAP2A,APP
SERPINC1_BACE1	1.03126973053648	9	0.114585525615164	SERPINC1,LRP1,MDK,STAT1,EN1,HOXA4,HOXA9,TFAP2A,BACE1
SERPINC1_C4BPA	1.06341587663877	11	0.0966741706035242	SERPINC1,LRP1,MDK,STAT1,TBX5,CDKN2A,E2F2,EZH2,HOXB13,AR,C4BPA
SERPINC1_CALR	0.869635427517727	3	0.289878475839242	SERPINC1,LRP1,CALR
SERPINC1_CAV1	1.02782418605743	9	0.114202687339714	SERPINC1,LRP1,MDK,STAT1,EN1,HOXA4,HOXB7,EGFR,CAV1
SERPINC1_DAB1	1.29803308766323	8	0.162254135957904	SERPINC1,LRP1,MDK,STAT1,TBX5,CDKN2A,CDK4,DAB1
SERPINC1_FZD1	0.869635427517727	3	0.289878475839242	SERPINC1,LRP1,FZD1
SERPINC1_GTPBP1	1.14020862984632	9	0.126689847760703	SERPINC1,LRP1,MDK,STAT1,EN1,HOXA4,HNF1A,LHX3,GTPBP1
SERPINC1_GULP1	1.06341587663877	11	0.0966741706035242	SERPINC1,LRP1,MDK,STAT1,TBX5,CDKN2A,E2F2,EZH2,HOXB13,AR,GULP1
SERPINC1_KAT5	1.02522210886751	7	0.146460301266787	SERPINC1,LRP1,MDK,STAT1,TBX5,CDKN2A,KAT5
SERPINC1_LPL	0.869635427517727	3	0.289878475839242	SERPINC1,LRP1,LPL
SERPINC1_MAPK8IP1	1.02782418605743	9	0.114202687339714	SERPINC1,LRP1,MDK,STAT1,EN1,HOXA4,HOXB7,EGFR,MAPK8IP1
SERPINC1_MAPK8IP2	1.02782418605743	9	0.114202687339714	SERPINC1,LRP1,MDK,STAT1,EN1,HOXA4,HOXB7,EGFR,MAPK8IP2
SERPINC1_MDK	0.869635427517727	3	0.289878475839242	SERPINC1,LRP1,MDK
SERPINC1_PDGFB	1.03126973053648	9	0.114585525615164	SERPINC1,LRP1,MDK,STAT1,EN1,HOXA4,HOXA9,TFAP2A,PDGFB
SERPINC1_SHC1	1.02782418605743	9	0.114202687339714	SERPINC1,LRP1,MDK,STAT1,EN1,HOXA4,HOXB7,EGFR,SHC1
SERPINC1_SKIL	1.0425145642797	7	0.148930652039957	SERPINC1,LRP1,MDK,STAT1,MMP9,COL4A2,SKIL
SERPINC1_ZNF8	0.869635427517727	3	0.289878475839242	SERPINC1,LRP1,ZNF8
SERPINC1_HSP90B1	1.06341587663877	11	0.0966741706035242	SERPINC1,LRP1,MDK,STAT1,TBX5,CDKN2A,E2F2,EZH2,HOXB13,AR,HSP90B1
SERPINC1_LTF	0.869635427517727	3	0.289878475839242	SERPINC1,LRP1,LTF
SERPINC1_PLAT	0.869635427517727	3	0.289878475839242	SERPINC1,LRP1,PLAT
SERPINC1_PLAUR	1.10846219469596	8	0.138557774336995	SERPINC1,LRP1,MDK,STAT1,TBX5,CDKN2A,E2F2,PLAUR
SERPINC1_PRKACA	1.14254770644913	8	0.142818463306142	SERPINC1,LRP1,MDK,STAT1,TBX5,CDKN2A,AURKB,PRKACA
SERPINC1_SERPINA1	0.869635427517727	3	0.289878475839242	SERPINC1,LRP1,SERPINA1
SERPINC1_SERPINE1	1.12477661526383	8	0.140597076907978	SERPINC1,LRP1,MDK,STAT1,TBX5,CDKN2A,E2F2,SERPINE1

Step 4: Obtaining the pathscore cutoff for the patient.

Code used: Rscript quantile.R TCGA_A6_0157_01A_sp_score.txt;

Output: TCGA_A6_0157_01A_sp.txt_score_quantile

This step calculates the quantiles of the normalised pathscore in the sample. We chose 0.01% as the standard cutoff to select the MutPaths in that sample based on the normalised pathscore.

Step 5: Obtaining the final set of mutpaths for the patient

Code used: bash get_mutpaths.sh TCGA_A6_0157_01A

This bash script filters the TCGA_A6_0157_01A_sp.tsv file to get the MutPaths based on the quantiles calculated in the previous step. This step results in a family of files that represents the MutPaths, mutated and DEG nodes in the MutPaths along with their interaction details. This serves as an input to the next step.

The following figure is the Cytoscape visualisation of the MutPaths with the nodes colored based on their foldchange values. The blue spectrum represents downregulated nodes while the red represents the upregulated nodes. The diamond-annotated nodes represent the mutated nodes present in the corresponding MutPaths.

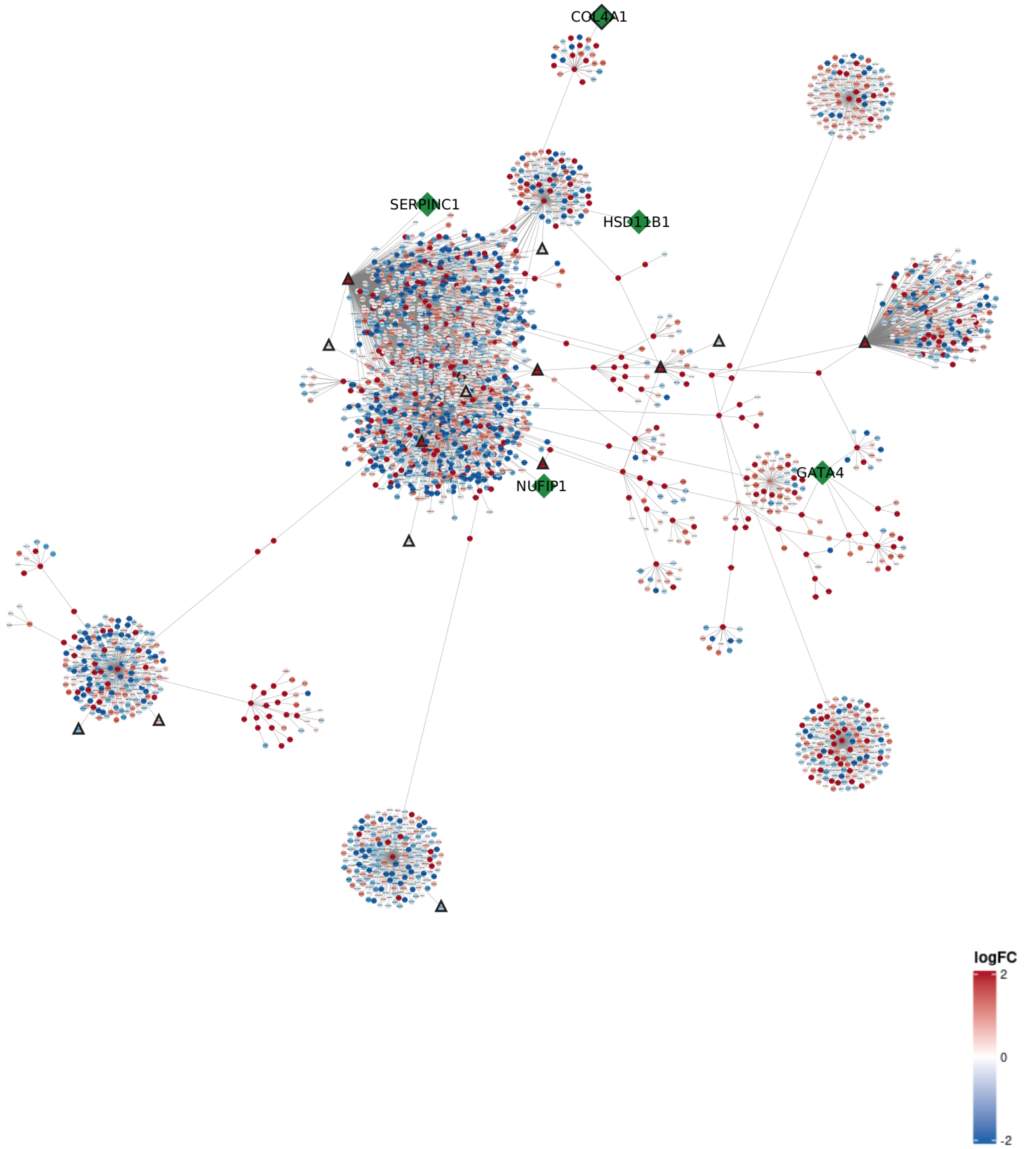

Figure 1: Network representation of the example MutPaths. The mutated genes are represented as green diamonds. The DEGs are represented in the blue-red spectrum based on their foldchange values where blue represents the downregulated nodes and red represents the upregulated nodes. The mutated nodes are represented as the larger node and annotated with their gene symbols in the network. The triangular nodes are the gold standard genes in glioblastoma.

Step 6: Obtaining the iPanels for the patient:

Code used: python props_maxcover.py TCGA_A6_0157_01A_toppath_int_wt.txt TCGA_A6_0157_01A_toppath_mut.txt TCGA_A6_0157_01A_deg.txt 70 TCGA_A6_0157_01A

This is the greedy approach employed to find the most influential mutated node that perturbs most of the downstream genes in the Mutpaths. This step also calculates the NetScore based on eq (4).
The following output screenshot represents the iPanel genes in the second row and all its associated DEGs (within a radius of 4) in the first row.

```
set(['TNC', 'EGFR', 'NCAN', 'MEIS1', 'OTX2', 'MYC', 'ASF1B', 'HOXA4',
'TOP2A', 'TFA2B', 'HOXB8', 'MCM10', 'HOXB3', 'HOXB4', 'HOXC10',
'MEOX2', 'PAX3', 'PRKCZ', 'UHRF1', 'E2F2', 'FN1', 'SHOX2', 'HOXA3',
'AR'])
set(['EGFR'])
```

The following Cytoscape visualisation represents the iPanel gene in the example sample along with its corresponding DEG nodes. The iPanel gene in this example is EGFR represented as a diamond node. The DEGs (significantly enriched nodes) are annotated with their gene symbols. The size of the nodes are directly proportional to their fold change values.

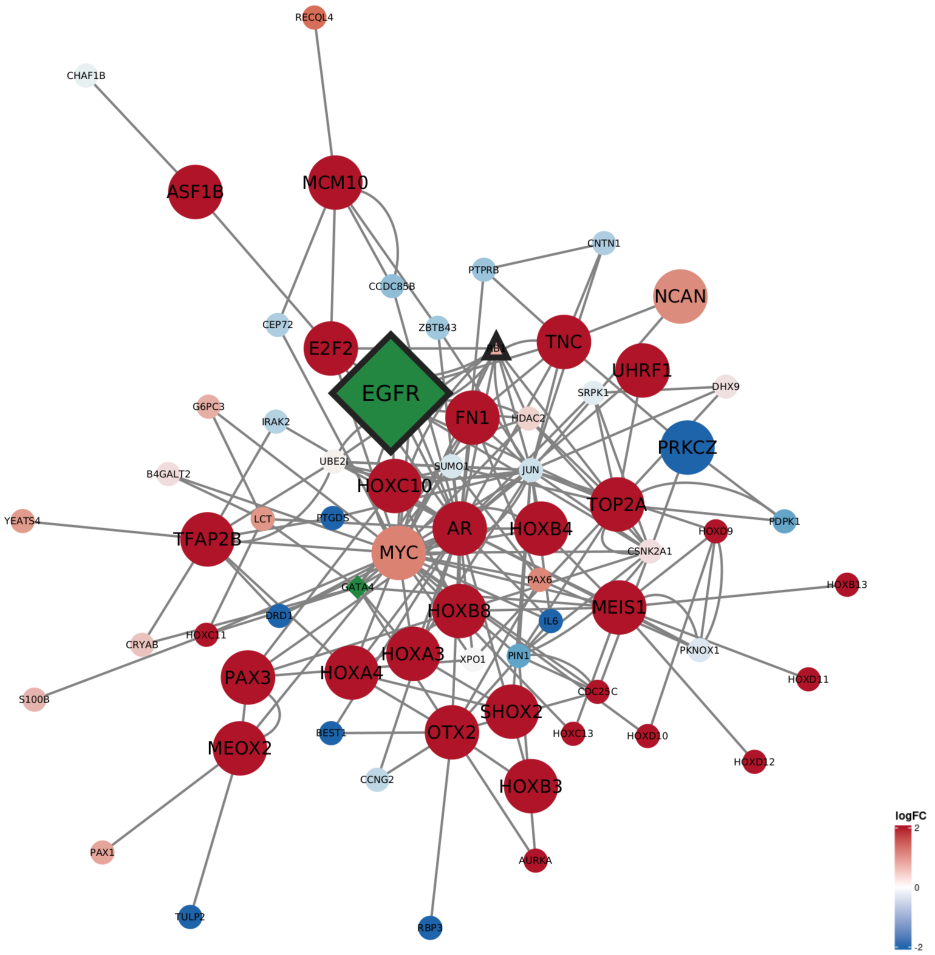

Figure 2: Network visualisation of the iPanel gene along with its downstream DEGs. The mutated genes are represented as green diamonds. The gold standard genes (excluding mutated ones) are represented by thick-bordered triangles. The DEGs are the larger circular nodes in the blue-red spectrum. The other connected nodes up to 4 hops are shown as smaller circles.

This tutorial ends with the identification of the iPanels. The next two modules, which compute PiRS and determine the actionability status, can be performed according to the description in the Methods section.

PiRS is computed using Eq. (8) which involves NetScore obtained from the previous step. The hazard ratio can be calculated using the conventional Cox proportional hazards model.

Supplementary Table 4 contains the actionability database used for the analysis. Researchers can simply compare the rows based on their iPanel genes to understand their actionability status.
